# The CNS/PNS Extracellular Matrix Provides Instructive Guidance Cues to Neural Cells and Neuroregulatory Proteins in Neural Development and Repair

**DOI:** 10.3390/ijms22115583

**Published:** 2021-05-25

**Authors:** James Melrose, Anthony J. Hayes, Gregory Bix

**Affiliations:** 1 Raymond Purves Bone and Joint Research Laboratory, Kolling Institute, Northern Sydney Local Health District, St. Leonards, NSW 2065, Australia; 2 Graduate School of Biomedical Engineering, University of New South Wales, Sydney, NSW 2052, Australia; 3 Sydney Medical School, Northern, The University of Sydney, Sydney, NSW 2052, Australia; 4 Faculty of Medicine and Health, The University of Sydney, Royal North Shore Hospital, St. Leonards, NSW 2065, Australia; 5 Bioimaging Research Hub, Cardiff School of Biosciences, Cardiff University, Cardiff CF10 3AX, UK; HayesAJ@cardiff.ac.uk; 6 Clinical Neuroscience Research Center, Departments of Neurosurgery and Neurology, Tulane University School of Medicine, New Orleans, LA 70112, USA; gbix@tulane.edu

**Keywords:** extracellular matrix, proteoglycans, agrin, perlecan, phosphacan, NG2 proteoglycan, Neuroglycan-C, lecticans, hyaluronan, neural repair and regeneration1

## Abstract

Background. The extracellular matrix of the PNS/CNS is unusual in that it is dominated by glycosaminoglycans, especially hyaluronan, whose space filling and hydrating properties make essential contributions to the functional properties of this tissue. Hyaluronan has a relatively simple structure but its space-filling properties ensure micro-compartments are maintained in the brain ultrastructure, ensuring ionic niches and gradients are maintained for optimal cellular function. Hyaluronan has cell-instructive, anti-inflammatory properties and forms macro-molecular aggregates with the lectican CS-proteoglycans, forming dense protective perineuronal net structures that provide neural and synaptic plasticity and support cognitive learning. Aims. To highlight the central nervous system/peripheral nervous system (CNS/PNS) and its diverse extracellular and cell-associated proteoglycans that have cell-instructive properties regulating neural repair processes and functional recovery through interactions with cell adhesive molecules, receptors and neuroregulatory proteins. Despite a general lack of stabilising fibrillar collagenous and elastic structures in the CNS/PNS, a sophisticated dynamic extracellular matrix is nevertheless important in tissue form and function. Conclusions. This review provides examples of the sophistication of the CNS/PNS extracellular matrix, showing how it maintains homeostasis and regulates neural repair and regeneration.

## 1. Introduction

The central and peripheral nervous system (CNS/PNS) extracellular matrix (ECM) has a unique composition and functional properties in the modulation of learning, memory, synaptogenesis and plasticity, and acts as a physical barrier. Like other tissues, the ECM plays a central role in the development of the CNS/PNS. Proteoglycans (PGs) are present in the ECM, are attached to cell membranes and are also intracellular components [1,2,3]. PGs have an extremely diverse group of core proteins and glycosaminoglycan (GAG) side chain complexity with critical supportive and guidance roles to play in the development of the CNS/PNS. These convey PGs with a range of cell-directive properties through interactions with guidance neuroregulatory, adhesive and structural glycoproteins and with cellular receptors, growth factors, chemokines and cytokines. Thus, PGs influence diverse cellular processes such as proliferation, differentiation, cellular migration and ECM assembly/remodelling in development and in tissue repair [4,5,6,7]. CNS/PNS astrocytes, neurons and glial cells synthesize PGs decorated with chondroitin sulphate (CS), keratan sulphate (KS), dermatan sulphate (DS), heparan sulphate (HS) and the human natural killer-1 (HNK-1) trisaccharide (SO_4_-3GlcAβ1-3Galβ1-4GlcNAc) [8,9,10,11,12,13,14]. Decoration of neural PG core proteins with these GAGs equips them with the capability of guiding embryonic nerve development and the formation of neural networks, as well as the development of the neural microvasculature [4,5,6]. Members of the lectican PG family interact with hyaluronan (HA) and tenascin-R to form specialized macromolecular ECM structures known as perineuronal nets (PNNs). These are neuroprotective, provide neural synaptic plasticity and are associated with cognitive learning [15]. Specialised basal structures in the synaptic motor neuron endplates have laminin-G containing HS-PGs that interact with muscle fibre basal structures containing dystroglycan and matriglycan, forming a functional interface in neuromuscular junction (NMJ) structures [14,16,17,18]. HS-PGs (agrin, collagen XVIII and perlecan) are also integral basement membrane components that maintain the integrity of the blood–brain barrier (BBB) [19,20].

Interaction of CS- and HS-PGs is essential in the generation of attractive or repulsive guidance cues by several cell surface neuroregulatory, cell adhesive and guidance proteins; these are covered later in this review. Movement of neurons occurs by two distinct processes: (i) extension and attachment of the leading process and (ii) translocation of the cell body. Neurons generate forces internally that extend the leading process as well as translocation of the cell body. A dynamic cytoskeletal system involving actin fibres, tubulin microtubules, intermediate microfilaments and nuclear envelope proteins generate forces that move the nucleus and centrosome to a central position, aligned with the axis of neuronal elongation, extends the cell process and co-ordinates cell polarization and the generation of focal adhesions. The ECM has central roles to play in neuronal migration. Abnormal processing of neural PGs can contribute to the development of a number of neurological disorders; thus, PGs are of interest as therapeutic targets. Fragmentation of neural PGs can also generate bioactive matricryptic modules which have proven to be useful in neural repair biology. An unusual feature of the CNS/PNS ECM is that it is mainly composed of GAGs, e.g., HA, but does not contain extensive fibrillar collagenous and elastic supportive networks. CS is a major component of lectican PGs, such as neurocan, brevican, versican, aggrecan and phosphacan [21]. KS, HNK-1 trisaccharide and DS are also constituents of some neural PG populations that possess important cell regulatory properties [22]; HNK-1 is also a component of neural structural and cell-adhesive glycoproteins (e.g., tenascin-C, tenascin-R and NCAM). HA is a major supportive component of the CNS/PNS ECM; its space-filling and hydrating properties are important in the compartmentalisation of specific brain regions and the maintenance of localised ionic microenvironments that are important for optimal cellular activity. HA also provides a highly hydrated matrix conducive to cellular migration and cell attachment during CNS/PNS neural network development. HA is a key component of PNNs, contributing to neuroprotective properties and to the establishment of neural synaptic plasticity that contributes to cognitive learning.

### 1.1. Glycosaminoglycans Convey Important Functional Properties to PGs and HA in the CNS/PNS ECM: Insights into Potential GAG Instructive Mechanisms in CNS/PNS Development and Repair

Sulphated GAGs represent major ECM components of the brain, constituting up to 60% of its mass during early embryonic development and 20% in the adult CNS/PNS. GAGs decorate PG core proteins, equipping them with important cell-regulatory and tissue functional properties [23,24,25]. PGs are abundant components of the CNS/PNS and include a family of large CS-PGs termed the lecticans, including aggrecan, versican, neurocan and brevican [26]. Phosphacan and NG2 PG are additional major large CS/KS/HNK-1-PGs with functional roles to play in the CNS/PNS [27,28]. A collection of small CNS/PNS PGs of varied structure and function also have important cell-regulatory properties. Under positive selection pressure, GAGs evolved in the glycocalyx surrounding all cells over an extended 500 million year period of invertebrate evolution to become cellular mediators with molecular recognition and information transfer properties [29]. These interactions are employed to regulate cellular behaviour during tissue development, remodelling and repair processes following trauma. Neurons are highly energetic cell types that utilise Na(+)/K(+)-ATPase pumps to generate energy. This process also generates chemical and electrical gradients across their cell membranes. This membrane polarization process is essential for cell signalling and regulates ionic balances that control cell volume. These processes are aberrantly controlled in neurological diseases [30,31,32]. Ions released by the neuron act as counter-ions to the sulphated GAG chains of PGs, which act as a local ECM ion-reservoir. Aggrecan is a large CS/KS-lectican PG which has a particularly high GAG content that may constitute up to 90% of its mass. Neuronally released Na+/K+ ions are taken up by the aggrecan GAG side chains as counter-ions. These influence the solvation volume of aggrecan and also attract water molecules that equip aggrecan as a polyelectrolyte gel contributing to its hydration properties in the CNS/PNS ECM through the Gibbs–Donnan equilibrium. The presence of negatively charged, impermeant proteins such as aggrecan alters the distribution of diffusible ions in neural tissue compartments and preserves tissue osmolality and electroneutrality. This property helps to counter osmotic shock and protects neurons following TBI. As shown later in this review, the lectican PGs do not have a uniform localisation throughout the brain ECM but are present as dense ECM-surrounding neurons termed PNNs, and are more diffusely distributed in the ECM between PNNs in the pre-frontal cortex and cerebellum. Neurons in the sub-ventricular zone also contain lectican PNNs but to a far lesser extent. Astrocytes also synthesize a large KS-PG termed Abakan [12]. While this PG is less extensively characterized than aggrecan, it has been localised to the margins of functional compartments in areas of the brain and its synthesis by astrocytes is upregulated following TBI [33]. Astrocytes also synthesize a small CS-PG termed astrochondrin [34]. This is an astrocyte cell surface PG which may facilitate interactive properties with the cell membranes of cerebral blood vessels and meningeal membranes. Astrochondrin carries L2/HNK-1 and L5 carbohydrate structures which may facilitate such interactions [34]. KS is also a highly interactive GAG and has unique functional properties [35]. KS is the most sensitive proton detection GAG and is a component of ultrasensitive electrosensory PG gels found in pore structures of elasmobranch fish species (rays, sharks), with the electrical signals detected interfaced with neural networks for signal interpretation [36,37,38,39].

GAGs can detect proton gradients and this process is considered to be fundamental to the evolution of life [40,41]. GAGs are electroconductive entities that participated in microelectronic events during the evolution of membrane polarization and membrane energetics that formed the basis of cell signalling [42,43,44,45,46]. Neurons are particularly sensitive to electrostimulation, with microelectronic events leading to polarization of the activated neuron cell membrane; however, this also contributes to membrane polarization in all cells, and forms the basis of cell signalling during cellular attachment, migration and transmission of signals from cell to cell not only during development but also in neural repair and functional recovery [47,48]. Thus, the GAG components of PGs participate in neurotrophic regulation of cellular movement in the development of neural networks and also in neural repair processes. A diverse collection of neuroregulatory molecules participate in these processes guided by cues from the ECM; these are discussed later in this review.

HA is the only non-sulphated GAG and is the most abundant GAG of the CNS/PNS ECM, and has important cell regulatory properties and contributes to the structural organization and maintenance of the hydration of the CNS/PNS ECM [49]. HA regulates the environment of cells and tissues and participates in many biological and pathophysiological processes, ranging from the regulation of neural stem cell differentiation to the proliferation, adhesion and migration of neural cell populations. HA can also fine-tune its viscoelastic space-filling properties in response to its microenvironment, e.g., pH, temperature and salt conditions, and maintains ionic gradients and niche micro-environments required for optimal cellular properties important for tissue function. The macroscopic response of HA to changes in its microenvironment involves alterations in hydrogen-bonding and hydrophobic interactions [50]. Investigation of HA interactions at the molecular level can be examined by two-dimensional infrared spectroscopy (2D-IR) [51] which, like 2D NMR, can resolve dynamic HA molecular bonding. Such molecular interactions involving hydrogen, hydrophobic, Van der Vaahl’s bonding or electrostatic interactions usually change on a time scale of picoseconds, and 2D-IR allows snapshots of these interactions to be made with femtosecond time resolution.

### 1.2. HS-PGs Have Diverse Roles in Early Neural Development

The ECM is a central functional component of all tissues and organs, making essential contributions to cellular regulation and the orchestration of tissue homeostasis to provide optimal tissue functional properties. From the earliest stages of embryonic development, diverse multiple ECM components regulate and fine-tune essential cellular processes that mould the form and function of the CNS/PNS [52,53,54]. Complex cooperative events with HS-PGs shape nervous system development [55]. BMP, Wnt and IHH signalling have key roles to play in neural tissue developmental processes, including early ectodermal tissue formation through regulation of the migration of neural crest cells [56,57]. Wnt also interacts with secreted factors from adjacent tissues to form these developmental gradients in embryonic neural tissues, and HS-PGs have key roles in the regulation of these interactive processes [53,54,58]. The Wingless morphogen gradient is established through the co-operative actions of Frizzled and cell surface HS-PGs, such as members of the syndecan family, which act as cellular receptors [58]. Sdc4 participates in the Wnt signalling pathway to regulate neural tube closure in mammalian embryos, and Sdc HS chains have important roles to play in this process [57]. Perlecan domain I binds BMPs, and Wnt and IHH proteins bind to perlecan domain II [13,59]. Perlecan has roles in the transport of these components to form developmental gradients in tissues; this is important in tissue development since Wnt and IHH are poorly soluble proteins. Perlecan regulates diverse cell signalling events in neural tissue development through interactions with the FGF and the VEGF growth factor family to regulate vascularisation and tissue development [59,60,61,62]. Neural cells have high metabolic demands and the brain microvascular system has essential roles to play in their nutrition, in what has been termed the neurovascular unit [60,62]. The essential roles of the coupled neurovascular system in neural health become apparent in neurodegenerative disorders and in ischaemic stroke when this system is disrupted [63,64,65,66,67,68]. Glypican-3 also has important roles in neural network development, controlling netrin-mediated axonal guidance [63]; the syndecans also act as cell surface receptors interacting with Slit-roundabout neural guidance proteins during neural network formation [53,54,69,70,71]. These are but two examples of how the HS-PGs finely tune neural tissue development [53,54].

### 1.3. The Diverse Roles of CS and DS-PGs in the Development of Neural Tissues

While HS-PGs have specific roles in early neural tissue development, CS is actually the most abundant GAG of the CNS/PNS ECM and CS-PGs also have important roles to play in neural tissue development [69]. Notochordal aggrecan has roles in the regulation of neural crest cell migration during development of the neural tube and notochord [70,71,72,73]. Notochordal aggrecan is modified with the HNK-1 trisaccharide and this has cell directive properties [9,74,75]. The sulphation patterns, epimerization and inversion in the structure of CS generates the related GAG, DS, also known as CS-B. CS and DS occur in significant amounts in the CNS/PNS and have tissue developmental properties [76,77]. CS/DS are co-polymers of GlcA-GalNAc and IdoA-GalNAc disaccharides. The GalNH_2_ of the former disaccharide is *O*-sulphated at C-4 or C-6. In DS, the GalNH_2_ moiety is almost exclusively sulphated at the C-4 position; however, minor proportions of L-idoA may be *O*-sulphated at C-2. This epimerization and inversion of D-GlcA to L-IdoA ranges from 1 to 99%; these residues are not regularly distributed along the CS/DS chain and occur in (i) blocks of ≥6 IdoA residues, (ii) alternating IdoA/GlcA units or (iii) as isolated IdoA units interspersed within regions of unmodified GlcA [78]. DS epimerase-1 and dermatan 4-*O*-sulphotransferase-1 form complexes that generate the long epimerized 4-*O*-sulphated blocks. The presence of idoA in DS results in a more flexible chain that can extensively explore spatial planar orientations, maximising binding opportunities with potential ligands. Thus, DS-containing PGs influence cellular migration, proliferation, differentiation, angiogenesis and cytokine/growth factor activities and CS-PGs also display similar diverse functional cell regulatory properties [8].

## 2. The Importance of Regulatory ECM and Cell-Associated PGs in CNS/PNS Formation

The development of the CNS/PNS involves the guidance of axons for considerable distances during the formation of neural networks, and this guidance process involves precise repulsive and attractive instructive cues generated by ECM PGs, and a range of neuroregulatory molecules (netrins, slits, ephrins, tyrosine kinase inhibitors, semaphorins, neuropilins and reelin) which interact with the neural cell populations, ECM and cell-associated PGs [77,79]. Neural cells are the longest cell type in the human body and have evolved some unique properties, with an ability to regulate membrane polarization along the length of the neuron. This is an important feature that initiates the co-ordinated migration of synaptic vesicles towards the synaptic gap where synaptic vesicles fuse with the de-polarised synaptic gap membrane. This releases a burst of neurotransmitters into the synaptic gap which are taken up by communicating neurons, resulting in signal transduction within the neural network. An intracellular KS-PG, synaptic vesicle PG-2 (SV2), has important transmitter and storage properties for synaptic vesicle neurotransmitters. Perlecan and laminin-1 also have prominent roles to play via basement membrane structures in the regulation of neural outgrowth in the embryo through provision of an environment conducive to cell adhesion and cell migration [80]. A novel form of notochordal aggrecan substituted with HNK-1 trisaccharide guides neural crest progenitor cells during early stages of embryonic neural development [74,75]. Integrins and growth factors also have prominent roles to play in neural tissue development, and these operate through interactions with several ECM PGs, and soluble ectodomain forms of transmembrane PGs such as phosphacan, neuroglycan-C and NG2 proteoglycan [81,82]. Specialised CNS ECMs known as PNNs, containing aggregate structures composed of members of the lectican CS-PGs, HA, tenascin and link protein, are important for neural protection, correct neural function and synaptic plasticity [83]. The lecticans are also focally up-regulated in the gliotic scar formation that stabilises CNS defects following injury. These PGs also produce a non-permissive environment in scars that inhibits neural outgrowth in animal models of brain trauma and spinal cord injury (SCI). Use of chondroitinase AC and ABC in these animal models shows that this inhibitory activity resides in the CS side chains of the lectican proteoglycans. Removal of these CS chains stimulates neuritogenesis and functional recovery of neural tissues [84,85]. Endogenous production of the CS-PG degrading ADAMTS-4 also promotes functional recovery in SCI by degrading CS-PGs in the scar [86]. Despite the generally inhibitory neural cues delivered by CS-A and CS-C, some CS-PGs containing highly charged CS disaccharides (CS-D, CS-E) or related DS disaccharides that can actually promote neural outgrowth and functional recovery [87,88,89]. These include populations of phosphacan, neuroglycan-C and NG-2 PG [90,91]. Furthermore, treatment of brain tissues in a rat model of experimental stroke with glypican or by enzymatic disruption of neurocan by intracerebral chondroitinase ABC improved cerebral anatomy, histological and functional recovery indices in the chronic phases of experimental stroke in rats [88]. Glypican and chondroitinase ABC increased neurite extension in cortical neuron cultures, with glypican increasing FGF-2 expression and chondroitinase ABC increasing brain-derived neurotrophic factor expression [88]. Some populations of phosphacan exhibit neurite outgrowth-promoting activity in embryonic rat and mouse hippocampal neurons through an oversulphated DS-D epitope embedded in the phosphacan CS side chains. This has been termed the DSD-1 epitope [89]. Neuroglycan-C is a transmembrane CS-PG member of the neuroregulin family [90] exclusively expressed in the CNS. A recombinant ectodomain of neuroglycan C has neuritogenic activity in cultured rat neocortical neurons.

Neural PGs are a diverse group of bioactive proteins. Armed with knowledge of their specific structures and the functional properties of particular neuroregenerative PGs, biomimetic approaches hold promise in repair biology applications to effect neural repair. CS-PGs act in conjunction with semaphorin 3A to direct interneuron migration away from the striatum in a tangential direction towards the cortex [91,92]. This contrasts with multiple CS/HS-binding chemoattractive factors which display variable roles during the complex interplay between attractive and repulsive cellular effects in the guidance of sensory axons to specific target sites [89]. CS-PGs also have important roles to play in the formation of synapses in PNNs and roles in neuronal plasticity, and the stabilisation and myelination of axons which maintain optimal electroconductive properties of neural networks [5].

During neural development, cells communicate with each other in a dynamic manner and with the ECM. This provides directive cues that co-ordinate neural and microvascular network formation [91,92,93]. Such cell–cell signalling and cell ECM interactions control cellular proliferation and differentiation, axonogenesis, cellular survival, migration and adhesion during neural tissue formation. Protein tyrosine phosphatases (PTPases) regulate protein tyrosine phosphorylation, providing regulatory roles of central importance in cell communication for tissue formation [94,95,96,97]. An extensive family of PTPases are produced by embryonic neurons and glia during tissue development [98]. Some PTPases (RPTPs) act as receptors for CS-PGs and HS-PGs that regulate neurite outgrowth and neuronal regeneration and also have roles in synaptogenesis [99].

## 3. Phosphatases, Kinases and Cell Signalling

Protein kinases and phosphatases catalyse the formation or hydrolysis of phosphate groups and the transfer of phosphate groups to proteins or hydrolysis of ATP to produce energy. Both of these enzyme groups thus act as phosphotransferases, but have opposite modes of action. Kinase genes constitute only 2% of the human genome but result in phosphorylation of >30% of all human cellular proteins [100,101,102,103,104]. The Eph receptors are the largest of the receptor tyrosine kinase (RTK) families, transducing soluble extracellular signals to the interior through ligand-induced activation of RTK kinase domains, but they can also mediate cell–cell communication through interactions with cell surface ephrin ligands on neighbouring cells. There are ten EphA and six EphB receptors in the human genome, that bind six G ephrin-A ligands and three transmembrane ephrin-B ligands, respectively [105,106,107,108]. EphA4 and EphB2 can also bind ephrins of a different class, and alternatively spliced forms of Eph receptors further extend the functional repertoire of this class of receptors.

### Receptor Protein Tyrosine Phosphatases and Receptor Tyrosine Kinases

**Figure 1:**
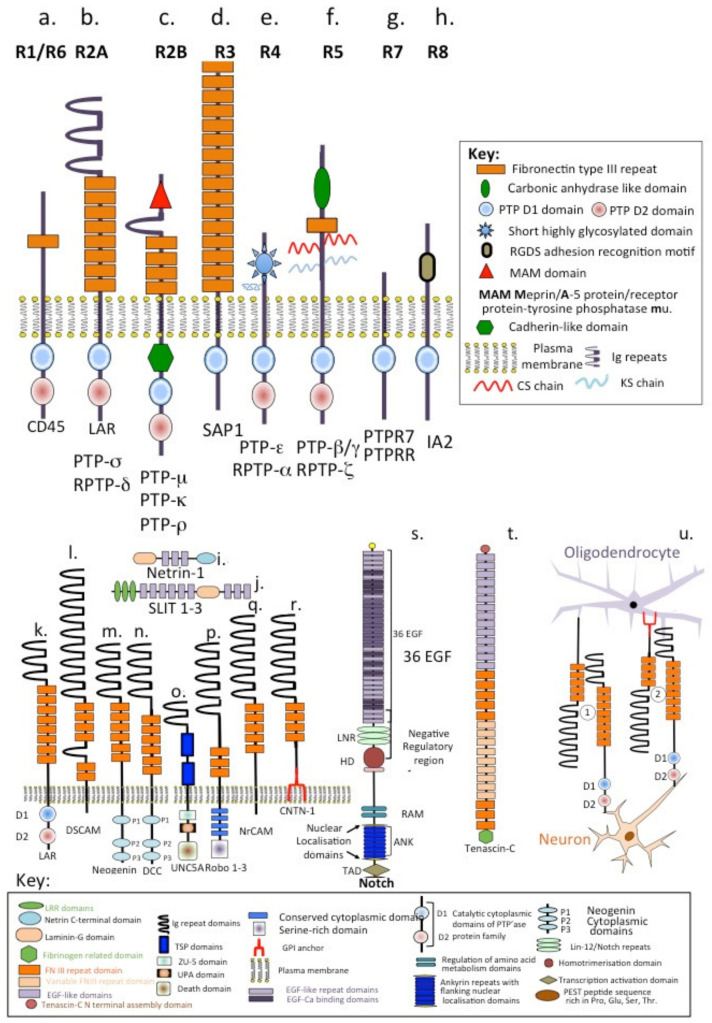
Schematic depictions of the structural organisation of the eight families of receptor protein tyrosine phosphatases (**a**–**h**) compared to neural cell adhesion molecules, neuroregulatory proteins and neural receptors, (**i**–**r**) Notch (**s**) and tenascin-C (**t**), and depiction of how interactions between receptor protein tyrosine phosphatases and neural cell adhesion molecules can facilitate oligodendrocyte–neuronal communication (**u**).

RPTPs constitute a large and structurally diverse superfamily of enzymes and have been sub-divided into eight sub families, R1/R6, R2A, R2B, R3, R4, R5, R7 and R8 (Figure 1a–h). There are 107 PTP genes in the human genome, of which 81 encode active protein phosphatases [109,110,111,112]. Leukocyte common antigen-related protein (LAR) tyrosine phosphatase (LAR-RPTP) and protein tyrosine phosphatase σ (PTPσ) are cellular receptors of HS-PGs and CS-PGs that regulate neurite outgrowth and neuronal regeneration. LAR and PTPσ are implicated in neuronal development, and strongly expressed in specific regions of the CNS, such as the sub-ventricular zone (SVZ). Binding of CS-PGs to LAR inhibits oligodendrocyte re-myelination [113,114,115] and negatively regulates oligodendrocytes in SCI and in amyotrophic lateral sclerosis (ALS) [116]. RTKs and RPTPs regulate signal transduction in many cell types by tyrosine phosphorylation and dephosphorylation, respectively [117]. PTPs include non-receptor-type PTPs and RPTPs, cell surface proteins that have intracellular tyrosine phosphatase activity and extracellular domains with sequence homology to NCAMs. LAR and PTPσ are widely expressed during neural development. Type IIa RPTPs (LAR, PTPσ and PTPδ) regulate synaptogenesis. Members of the connectin (CNTN) family of NCAMs also have roles in the formation and maintenance of the CNS. Inappropriate expression or interactions of CNTNs is linked to mental retardation and neuropsychiatric disorders such as autism. Five of the six CNTNs bind homologous PTP-γ and PTP-ζ receptors. Binding between RPTP-ζ and CNTN1on oligodendrocyte precursor cells inhibits their proliferation and promotes their development into mature oligodendrocytes [118].

## 4. Netrins

Netrins, named after the Sanskrit word “netr”, which means “one who guides”, are soluble axonal guidance proteins of similar structure to the laminin α and γ chains [119,120,121,122] (Figure 1i). Netrins are a family of proteins that direct cell and axon migration during development of the PNS/CNS. Three secreted netrins (netrin-1, -3 and -4) have been identified in mammals, in addition to two GPI-anchored membrane-bound netrins, (netrin-G1 and G2). The secreted netrins are bifunctional proteins, acting as attractants for some cell types and like the Slits as repellents for others (Figure 1j) [123,124,125].

## 5. Comparison of the Structural Organisation of LAR, DSCAM, Neogenin, DCC, UNC5A, Robo, NrCAM, CNTN-1, NOTCH and Tenascin-C

Down syndrome cell adhesion molecule (DSCAM), a member of the immunoglobulin superfamily of cell adhesion molecules, has multiple functions providing attractive or repulsive cues depending on the ligands it interacts with (Figure 1l). Netrin-DCC or neogenin complexes are attractive receptors, while netrin-UNC-5-related protein complexes have repellent properties [123,124,125,126] (Figure 1m–o). The Robo 1–3 receptors are critical regulators of neurons that cross the midline during network formation (Figure 1p). Comparison of the structural organisation of the aforementioned receptors and the cell adhesion molecules LAR, NrCAM and CNTN-1 shows a recurring theme between them. Immunoglobulin repeats and FNIII repeat modules are prominent features in all of these, except UNC5A (Figure 1k–r), while each has specific intracellular features reflecting their abilities to induce cell signalling or not. The structures of two highly interactive proteins, NOTCH and tenascin-C, are also shown for comparison (Figure 1s,t). These both have prominent EGF repeat regions and tenascin-C has additional multiple FNIII and variably spliced FNIII modules reflecting its interactive properties. NrCAM–LAR (1) and CNTN-1–LAR (2) interactions facilitate communication between oligodendrocytes and neurons (Figure 1u).

### Cell and ECM Interactive Functional Modules Are Also Present in CNS/PNS PGs

CNS/PNS ECM PGs also contain a number of protein and cell interactive modules in their respective core proteins which facilitate functional interactive properties that synergise or complement their GAG side chain-mediated interactions. The immunoglobulin A and B modules in the N-terminal globular domains of the lecticans facilitate their interactions with HA and macroaggregate network formation in PNNs. Immunoglobulin repeat modules in perlecan domain IV have cell-binding properties, while the laminin-G modules in perlecan domain V and C-terminus of agrin promote interactions that stabilise the BBB and NMJ. NG2-PG also contains C-terminal Lam-G domains which help localise this PG in the ECM following its release from the cell surface by proteases. C-terminal lectin modules of the lecticans provide cell interactive properties with roles in the regulation of cell migration. The central LRR modules of the SLRP family convey protein interactive properties. The shed ecto-domain of phosphacan contains terminal carbonic anhydrase modules that have protein interactive properties and fibronectin type III repeat modules that facilitate phosphacan interactions with NCAMs through heterophilic interactions at the neural cell surface. Astrocytes synthesise a small cell surface CS-PG, astrochondrin, which displays L2/HNK-1 and L5 carbohydrate motifs that facilitate interactions with membrane proteins in the cerebral microvasculature and membranes of the meninges. The core protein of neuroglycan-C on the neuron cell surface contains EGF repeat modules that facilitate cross talk of neurons with other CNS cell populations, while its CS side chains act as a receptor for HS-binding growth factors that promote neuritogenesis and neuronal cell migration.

## 6. The Semaphorins

ECM molecules that inhibit CNS development fall into three main categories: (i) axonal guidance molecules (e.g., semaphorins, ephrins and netrins), (ii) myelin-derived inhibitors (Nogo, MAG and OMgp) and (iii) CS-PGs (lecticans, phosphacan and NG2). Neural growth-promoting ECM molecules include cell adhesion molecules, and neurotrophic factors. Attractive and repulsive cues both critically guide axonal development to their physiological target sites in the CNS. Guidance molecules of importance in CNS development include the semaphorins, netrins, slits and ephrins. During neural development, spatiotemporal patterns of repulsive and attractant cues in the vicinity of the growth cone co-ordinate neurite outgrowth. The injured CNS axon does not regenerate due to outgrowth inhibitory proteins expressed in and around the lesion scar. Myelin-derived inhibitors, CS-PGs and tenascin-R all display inhibitory properties with regard to neural repair. Class 3 semaphorins and their receptors, neuropilins and plexins [127,128], which display neurite growth-inhibitory cues during axonal elongation and neural development, are also expressed in neural scars following trauma to the CNS, apparently contributing to the lack of repair of such lesions that prevent functional recovery of CNS neurons.

**Figure 2:**
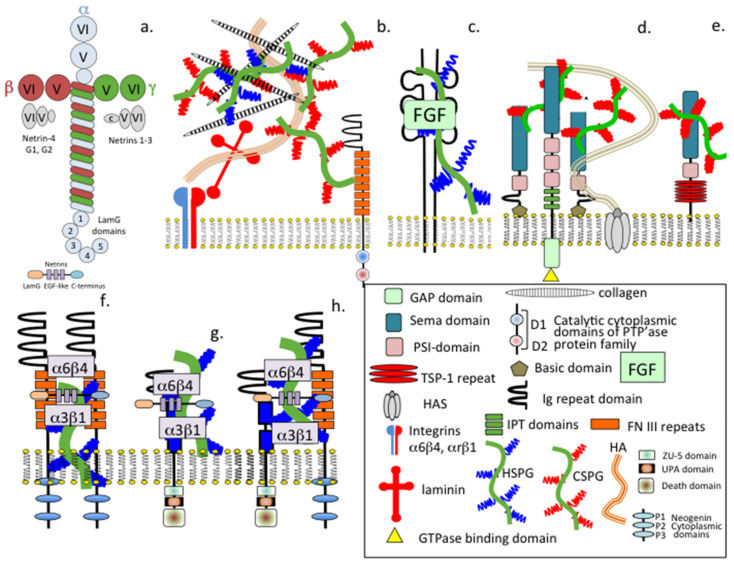
Schematic depiction of the structural organisation of generic laminin (**a**) and the related netrin neuroregulatory proteins (**b**–**h**) and depiction of how HS-PGs and CS-PGs interact with representative cell adhesion molecules and cellular receptors that promote neuron proliferation through growth factors sequestered by these proteoglycans (**b**,**c**) or facilitate attractive or repulsive neuroregulatory cues by semopharin3A-neuropilin (**d**) or semaphorin 5A (**e**). HS-PGs also participate in integrin-mediated dimerization interactions with cell adhesion molecules at the cell surface (**f**) and in interactions with netrin-1 and UNC-5A (**g**) and UNC5A-Neogenin/DCC netrin interactions (**h**).

HS-PGs interact with members of the RPTPs, FGFRs, semaphorin-neuropilin, neogenins and DCC at the cell surface to effect neuronal regulation. Some members of the RPTPs act as PG receptors. For example, binding of HS-PGs to LAR promotes neural outgrowth. HS-PGs act as co-receptors for the FGFs and facilitate FGF–FGFR interactions that lead to neuron proliferation. Members of the semaphorins and neuropilins also interact with CS-PGs at the neuron cell surface. However, CS-PGs inhibit neuritogenesis (Figure 2). Besides acting as signalling molecules providing axonal guidance cues during neural development, the semaphorins also have roles in immune regulation, angiogenesis and invasive tumour growth [129,130,131,132,133]. The main functional receptor for semaphorins are the large single pass transmembrane plexins. Interaction of the semaphorins and plexins results in cytoskeletal remodelling, integrin-mediated adhesion and neuron migration. Semaphorins can also undertake “reverse signalling” in which rather than acting as ligands they act as receptors and initiate a signalling response through their own cytoplasmic domains [129,130]. Thus, the semaphorins have multifaceted properties in neurodevelopment and in the pathological CNS following injury or disease [131,132,133,134,135,136,137].

Perlecan facilitates transmembrane repulsive semaphorin-mediated guidance cues that direct neural development and axonal development and co-ordinates longitudinal neural tract formation. The meninges that surround and protect the CNS also secrete soluble repulsive and attractive neuroregulatory guidance proteins that regulate directional migration and longitudinal growth of neurons and axons in spinal cord development. Fibrogenic cells are restricted to vascular and meningeal areas of the CNS. Following spinal cord injury, these cells secrete PGs that regulate neuro-inflammation, and the fibrotic repair response and fibrotic scar formation [138,139,140]. Perlecan regulates this fibrosis process [141]. In the injured CNS, when damaged neurons do not induce neural tissue regeneration, scar-forming endothelial cells, inflammatory immune cells, stromal fibroblasts and astrocytes lay down a fibrotic scar in brain and spinal cord lesions. This scar stabilises damaged tissue areas, but it also restricts neural regeneration and functional recovery [142,143,144].

**Figure 3:**
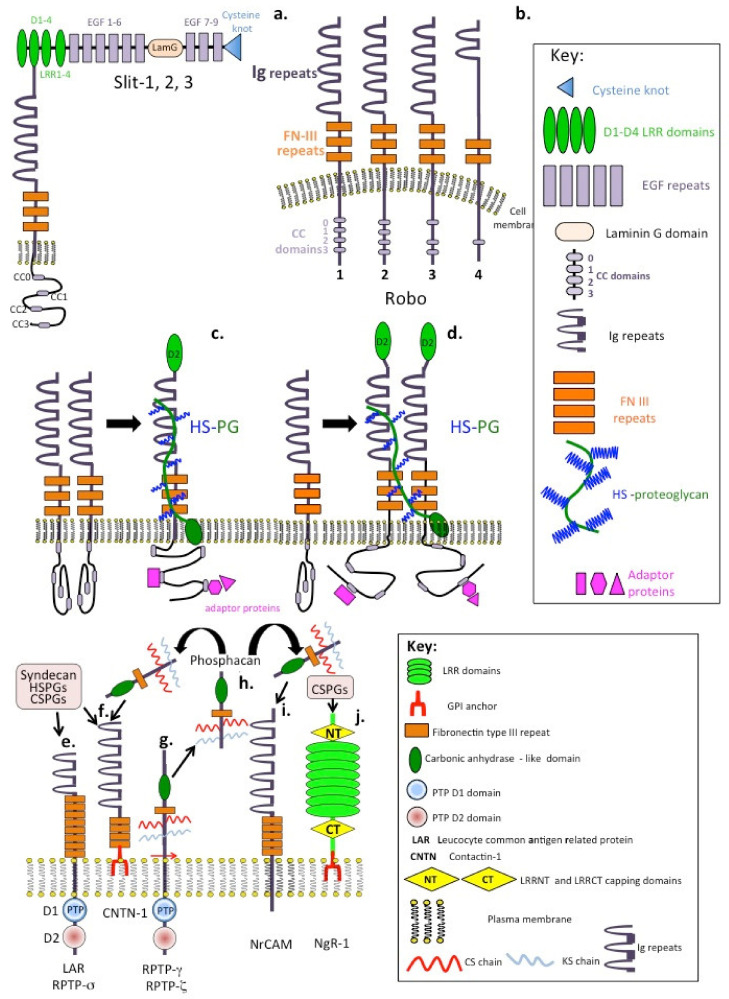
Schematic depiction of the modular structural organisation of Slit-1, 2, 3 and the Robo-1, 2, 3, 4 receptors. Depiction of the structural reorganisation and interaction with HS-PGs that leads to activation of the Slit proteins and re-organisation of cytoplasmic adaptor proteins in this process (**a**–**d**). Depiction of the modular organisation of protein tyrosine phosphatase receptors (**e**), contactin (**f**), the RPTP-ζ preform of phosphacan (**g**) and the released ectodomain, phosphacan (**h**) and NrCAM (**i**) NgR-1 Nogo receptor (**j**) that both act as PG receptors with binding of CS-PGs inducing an inhibitory signal that prevents neurite outgrowth and neural proliferation, while HS-PGs promote neural activity including neural outgrowth.

Slit is a large secreted 200 kDa LRR protein that provides important guidance cues in the developing nervous system [145,146]. The secreted Slit glycoproteins and their Roundabout (ROBO) receptors have major axon guidance roles in cell repulsion that prevent commissural axons from migrating inappropriately during the assembly of the nervous system but also have recently discovered roles in neurogenesis and angiogenesis [147,148,149,150,151,152,153,154,155]. Vertebrates contain four different Robos and three Slits: Robo1, Robo2, Robo3/Rig1 and Robo4; and Slit1, Slit2 and Slit3. An extensive literature demonstrates the conserved roles conveyed by the Slit–Robo system in the guidance of axons during the development of the nervous system throughout vertebrate evolution [153]. Robo1 and Robo2 signal repulsive cues in response to Slit preventing inappropriate midline crossing of axons during neural development. However, Robo3 promotes midline crossing. Signalling by Slit requires two receptors, Robo transmembrane proteins and HS-PGs [154] (Figure 3a–d). Robo is one of the most studied neural receptors and has critical roles directing axonal migration. Glial cells migrate over long distances to form stable interconnections with neurons [156,157,158,159,160,161]. The netrin and semaphorin families have important guidance roles which control glial cell migration [162,163,164].

## 7. Cell Surface Receptors That Bind CS- and HS-PGs to Regulate Neuritogenesis

Some members of the RPTPs, NrCAMs and related CNTNs act as PG receptors, binding CS- and HS-PGs at the cell surface. These include LAR, CNTN-1 and NrCAM (Figure 3e,f,i), while RPTP-ζ is a PG that undergoes proteolytic processing, releasing its ectodomain as a soluble PG (phosphacan) that also interacts with these receptors (Figure 3g). Ngr-1 also interacts with CS-PGs. Binding of CS-PGs to these receptors results in an inhibitory signal preventing neural outgrowth, while HS-PGs generally promote neuritogenesis. CS normally inhibits regeneration in the adult CNS. However, the CS-binding growth factor pleiotrophin (PTN) is also expressed at high levels in the developing CNS and can reverse the inhibitory effect of CS chains on neurite outgrowth [165]. This effect is achieved by the binding of PTN to cell surface glypican-2. This also abrogates CS ligand binding to the inhibitory receptor RPTPσ. Thus, PTN increases dendrite regeneration in the adult cerebral cortex and axonal regeneration in the injured adult spinal cord [166]. PTN also increases neural proliferation and neurite outgrowth in cultured neurons. Furthermore, PTN knockout mice exhibit severely impaired auditory responses. PTN is also decreased following acoustic trauma and aging, indicating a potential role for PTN in neuroregulatory processes in inner ear development and function. Upregulation of CS-PGs within the glial scar and PNN creates a barrier to axonal regrowth and sprouting. RPTPσ, LAR and the Nogo receptors Ngr1 and Ngr3 bind CS-PGs and this inhibits neuronal recovery following SCI. Interaction of CS-PGs with RPTPσ results in dystrophic growth cone collapse. Systemic delivery of a function-blocking peptide has been used to modulate RPTPσ interactions with CS-PGs to promote neuronal recovery following SCI. Inhibition of cortical dendritic growth by CS-PGs, can also be counteracted by reelin signalling [166]. Reelin is an extracellular signalling protein that regulates the alignment of radial neurons and rescues neurite outgrowth in the presence of CS-PGs. Furthermore, chondroitinase injections into *reeler* mice (reelin knockout) explants results in increased dendritic growth and neuron numbers in the marginal zones where reelin is highly expressed. Activation of the serine threonine kinase Akt is required for the stimulation of reelin-dependent dendritic growth; however, CS-PGs induce Akt dephosphorylation, an effect that can be counteracted by reelin in vitro and in vivo [167]. Epac is a guanine nucleotide exchange factor for Rap1 and represents an intracellular target that is activated by cAMP [167]. Epac2 transforms the post-lesional inhibitory environment following SCI to promote axonal outgrowth in a model of SCI [168]. Epac2 activation using a specific soluble agonist (S-220) significantly enhanced neurite outgrowth of postnatal rat cortical neurons and markedly overcame the inhibition by CS-PGs and mature astrocytes on neuron growth. Epac2 also enhances neurite outgrowth in vitro, even in the presence of an inhibitory environment rich in CS-PGs and offers therapeutic potential in the treatment of traumatic injury to the CNS [169].

While CS-A- and CS-C-substituted lectican PGs inhibit neural outgrowth and functional neuronal recovery in gliotic scars following SCI and TBI, some CS-PGs, such as phosphacan [170], NG2 (CSPG4) [171] and neuroglycan C (CSPG5) [172], decorated with CS-E can reverse this inhibitory signalling to promote neuritogenesis and functional recovery of neural tissues.

## 8. Nogo

**Figure 4:**
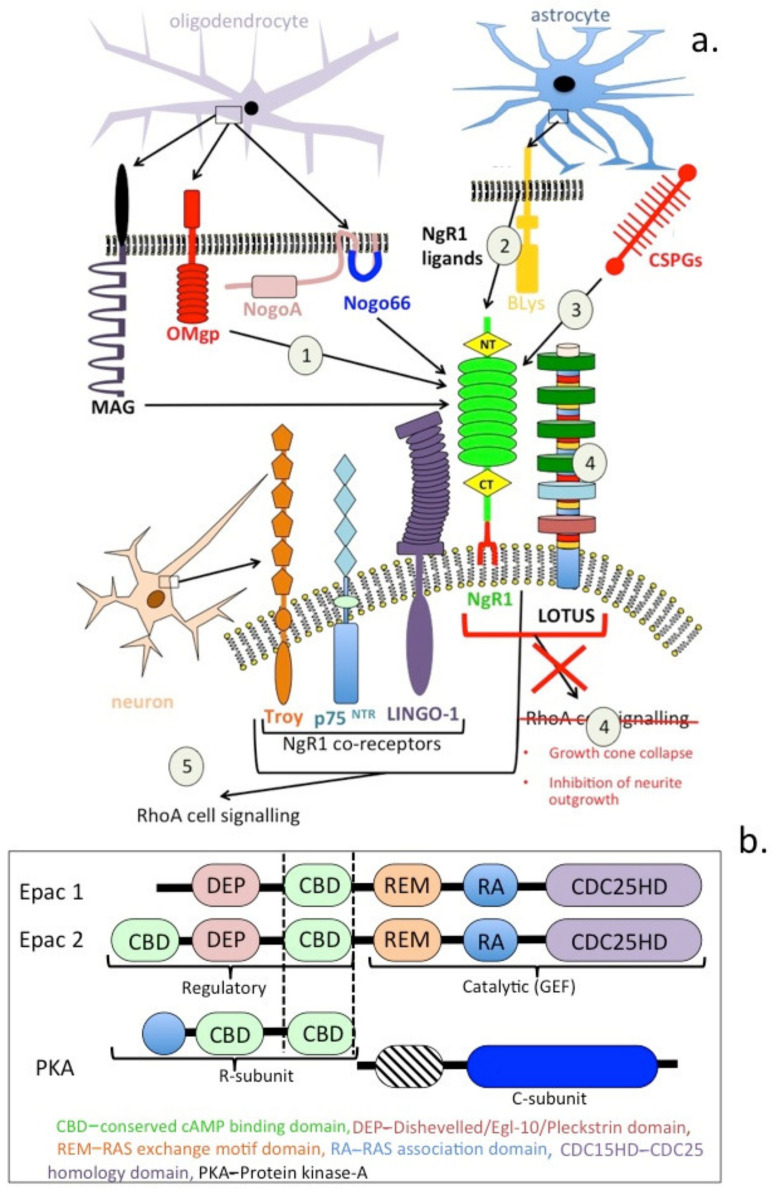
Schematic representation of the NgR1 Nogo receptor and Nogo co-receptors (Troy, p75 ^NTR^ and Lingo-1) expressed by neurons, which collectively produce an inhibitory, proliferative and neurite extension signal upon binding of AGIs such as MAG, OMgp, NogoA and Nogo 66 (**a**) produced by oligodendrocytes (1) and BLys (2), or CS-PGs (3) produced by astrocytes, with the cytoplasmic domains of the co-receptors promoting RhoA signalling (**a**). Depiction of the LOTUS NgR1 antagonist which blocks RhoA signalling (4), growth cone collapse and the inhibition of neurite outgrowth induced by AGIs (5). Modular structure of Epac1 and 2 (cAMP-regulated guanine nucleotide exchange factors-1, 2) (**b**) which mediate the action of cAMP and protein kinase A. Epac2 transforms the post-lesional inhibitory environment following SCI to an environment conducive to axonal outgrowth and neural proliferation in a model of SCI.

The adult CNS rarely recovers from injury; this is due to a number of axonal growth inhibitory proteins (AGIs) derived from myelin, such as Nogo protein [173], myelin-associated glycoprotein (MAG) [173] and oligodendrocyte myelin glycoprotein (OMgp) [174]. Glial cells also produce AGIs such as CS-PGs, while astrocytes produce a B lymphocyte stimulatory AGI which is a member of the TNF superfamily [175]. The AGIs bind to NgR1, resulting in growth cone collapse and the inhibition of neurite outgrowth activity (Figure 4a, 1–5). Thus, Nogo-A has major roles to play in neurite growth-inhibitory and regenerative effects exerted by myelination in the mammalian brain and spinal cord following traumatic injury [176]. High amounts of intracellular Nogo in neurons and interactions with β-secretase indicate Nogo may also regulate amyloid precursor protein (APP) processing. Nogo has structural roles in the ER and nuclear membrane that regulate cell survival and apoptosis. Nogo-A, OMgp and MAG are expressed by oligodendrocytes, and they inhibit axonal growth upon binding to NgR1–3 [177]. An antagonist to the Nogo receptor, Lateral olfactory tract usher substance (LOTUS) has also been described and shown to promote functional recovery in traumatised neural tissues, accelerating neuronal plasticity after spinal cord injury and cerebral ischemia in mice [178,179].

Nogo is a CNS-specific inhibitor of axonal regeneration and a regulator of neuron precursor cell migration and neuritogenesis [180]. Nogo A KO mice display schizophrenia-like behaviour similar to psychiatric disorders associated with mutations in genes encoding Nogo or NgR1 [181,182,183,184].

The Nogo receptor NgR1 is a 473-amino acid LRR protein containing eight LRRs flanked by N- and C-terminal disulphide stabilised-capping regions, a C-terminal stalk and GPI anchorage. All eight of the LRR domains, and the cap regions, are required for Nogo binding and for inhibitory signalling activity. NgR1 binds MAG and OMgp with high affinity [185]. NgR1 is a GPI-anchored receptor and requires a co-receptor to transduce signals to the cytosol. Nogo co-receptors include p75 [186], Troy [187,188] and LINGO [189]; they facilitate Nogo–NgR1 signalling and activation of RhoA [190]. LOTUS, a Nogo antagonist, prevents interactions between Nogo and its co-receptors [191,192]. Inhibition of NgR1 activity promotes functional recovery in animal models of traumatic injury to the CNS [193,194,195]. NEP1–40 peptide, a Nogo-66 antagonist, has also been shown to promote axonal regrowth in a rat SCI model and improves motor function [194]. Triple KO of Nogo/MAG/OMgp in mouse models shows greater axonal regrowth and improved motor functions following SCI [195].

Lateral olfactory tract usher substance (LOTUS) was originally identified as cartilage acidic protein-1B (Crtac1B), a marker protein that differentiated human chondrocytes from osteoblasts and mesenchymal stem cells in culture [196,197]. Two different Crtac1 transcripts were subsequently shown to be expressed in lung and brain and to possess antagonistic activity against the neuroinhibitory activity conveyed by Nogo and its co-receptors over axonal growth [198,199,200]. Cratc1B, C was subsequently named endogenous neural circuit promotion factor, LOTUS [199]. LOTUS is expressed in healthy neurons and promotes assembly of axonal bundles by inhibiting Nogo–NgR1 interactions, completely suppressing the growth cone collapse and neurite outgrowth inhibition promoted by NgR1. Inhibition of neurite outgrowth by CS-PGs supplied by astrocytes in scar tissues following CNS trauma is also explained by the CS-PGs acting as ligands for NgR1, promoting its inhibitory activity (Figure 4). LOTUS stimulates neuronal regeneration following ischemia in a middle cerebral artery occlusion model in mice [200].

## 9. Reelin and Its Directional Control of the Development of Radial Neurons

**Figure 5:**
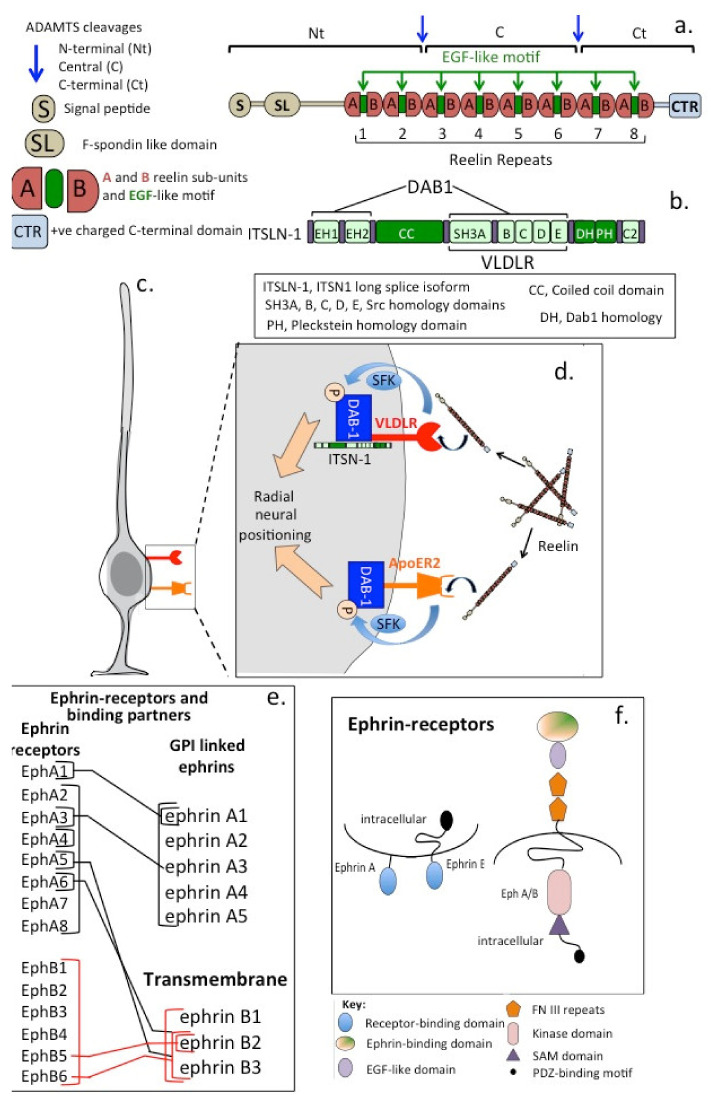
Schematic depiction of salient features of the structural organisation of the radial neuron regulatory glycoprotein reelin (**a**) and its regulatory protein DAB-1 (Disabled-1) (**b**) which have guidance roles that control radial neuron growth and orientation signalling through the very low-density lipoprotein receptor (VLDLR) and apolipoprotein E receptor 2 (ApoER2) (**c**,**d**). The complexity of ephrin–ephrin receptor signalling showing ephrin receptors and the GPI anchored and transmembrane ephrin signalling combinations (**e**). Structural representation of the structural organisation of the ephrin receptors (**f**).

Reelin (Reln), a 440 kDa secreted ECM glycoprotein with 18 potential N-glycosylation sites (Figure 5), regulates neuron migration in the foetal and adult brain by controlling cell–cell interactions [201,202] to regulate synaptic plasticity [203,204]. Reln stimulates dendritic process development and neuroblast migration in the subventricular and subgranular regions of the brain during neurogenesis [205,206]. Heterozygous reeler mice [207,208] that express reduced levels of Reln are anatomically normal but display behavioural and physiological abnormalities [207]. Reln is implicated in the pathogenesis of a number of brain diseases. Expression of Reln is significantly lower in schizophrenia, bipolar disorder and in major depression and may also have roles in Alzheimer’s disease (AD), temporal lobe epilepsy and autism [209,210,211]. The primary phenotype in heterozygous reeler mice is an abnormal neuronal positioning in the developmental CNS and behavioural traits related to psychotic disorders; however, few neuroanatomical defects are evident [212]. The primary function of Reln is the regulation of corticol development and neuronal cell positioning [213]. Reln acts through two receptors expressed by neurons and glial cells, very low-density lipoprotein receptor (VLDLR) and ApoER2 [213,214,215]. Reln is essential for brain development, induces the Notch-1 signalling cascade and results in neuroprogenitor cells assuming a radial orientation. Reln and cell surface adhesion receptors such as α3β1 integrin regulate neuronal migration and positioning in the developing cerebral cortex [216]. α3β1 integrin binds to the N-terminus of Reln at a site distinct from the VLDLR/ApoER2 binding sites. Dab1, a member of the reelin signalling pathway, complexes with a cytoplasmic region of beta1 integrin in a reelin-dependent manner, contributing to neuronal placement in the cerebral cortex [217]. Loss of the signalling scaffold intersectin 1 (ITSN1) adapter protein in mice also leads to defective neuronal migration by ablating reelin hippocampal activity [218]. ITSN1 interacts with reelin receptors, as evidenced by prominent defects in neuronal migration and radial glial distribution in the hippocampus and cortex in double-KO mice lacking ITSN1 and ApoER2 [219]. ITSN1 associates with the VLDLR and its downstream signalling adaptor Dab1 to facilitate reelin signalling. Thus, ITSN1 is a component of reelin signalling in the VLDLR–Dab1 axis, directing neuronal migration in the cortex and hippocampus and augmenting synaptic plasticity [218,219]. These observations in reelin mice have been confirmed in a number of human studies [220]. Reduced reelin mRNA levels have been observed in schizophrenia patients [221,222,223] and in post-mortem studies in the hippocampus and cerebellum [223], basal ganglia [224] and cerebral cortex [225,226]. Reelin is cleaved by MMPs in vivo at two sites located after domains 2 and 6, between repeats 2 and 3 and repeats 6 and 7, resulting in the production of three fragments [227]. Catabolism of reelin, however, does not appear to decrease its bioactivity.

### Reduction in Reelin and GAD67 Expression Is Associated with Neurological Disorders

Reelin promotes hippocampal neuron dendrite development [228,229] and counters the inhibitory signal imposed by CS-PGs on neurite outgrowth. Reelin mRNA levels may reach a 50% reduction in some neurological disorders, accompanied by reduced expression of glutamic acid decarboxylase (GAD-67) [222]. γ-amino butyric acid (GABA) is produced by GABAergic neurons and is the principle inhibitory neurotransmitter in the adult human brain reducing neuronal excitability. Binding of GABA to transmembrane receptors in the plasma membrane of inhibitory neurons opens ion channels, causing an influx of chloride ions or efflux of potassium ions, resulting in a negative change in the cell membrane transmembrane potential, and may cause hyperpolarization. Inhibition of this process can lead to psychosis and seizures. While GABA is an inhibitory signal in the adult brain countering feelings of anxiety and fear, it also acts as an excitatory signal during neural network development, acting in an autocrine or paracrine fashion. GABA regulates neuroprogenitor cell proliferation [230], migration [231] and differentiation [232,233], elongation of neurites [234] and formation of synapses [235]. GABA is primarily synthesized from glutamate by glutamate decarboxylase, which occurs as 65 and 67 kDa isoforms (GAD65 and GAD67). GAD67 has widespread activity throughout the neuron, while GAD65 activity is primarily focused to nerve terminals. This partly explains why reduced Reln levels impact on neurological disorders such as schizophrenia [209,210]. The Reln receptors ApoER2 and VLDLR are members of the LDL receptor family, which are all also receptors for apolipoprotein E (ApoE). ApoE occurs as three iso-forms (E2, E3 and E4). ApoE4 is a primary genetic risk factor for late onset AD, implicating ApoE receptors in the pathogenesis of AD [236,237].

## 10. Ephrins

Ephrin receptors are the largest known subfamily of RTKs (Figure 5e,f). Ephrin ligands (ephrins) and ephrin receptors (Ephs) are membrane-bound proteins [238]. Ephrin–Eph receptor cell signalling is dependent on direct cell–cell contact. Such interactions direct embryonic neural development and regulate the development of axonal growth cones [239]. Subclasses of ephrin-A and ephrin-B are categorised on the basis of their structures and cell membrane linkages (Figure 5e,f). Ephrin-As are anchored to membranes by a GPI linkage and do not have a cytoplasmic domain. Ephrin-Bs are attached to membranes via a single transmembrane domain containing a short cytoplasmic PDZ-binding motif (Figure 5f). Ephrin–ephrin receptor signalling is critical to cell–cell mediated migration of neuronal axons to their targeted destinations [238,239]. Eph–ephrin activation is associated with decreased growth cone survival and the repulsion of migrating axons but can also promote cellular migration. Ephrins promote angiogenesis in normal neural tissues but also in pathological neural tissues and may promote tumour development.

## 11. ECM Components Influence Cell Migration during Neural Development and CNS Repair

The CNS ECM and secreted PGs both influence cellular migration, attachment and proliferation during development and in wound repair responses [240]. In cases of traumatic injury to the CNS, axonal regeneration is strongly impeded by scarring at the impact site with the deposition of CS and KS-PGs providing inhibitory cues at the defect site [241]. Many studies show that up-regulation of scar PGs is influenced by the inflammatory environment of the scar through the secretion of cytokines and chemokines, by infiltrating astrocytes, macrophages and microglia. Members of the cell surface Sdc PG family are also upregulated around the scar and these undergo shedding of their ectodomains, further contributing to the inflammatory scar milieu. Moreover, TGFβ-induced synthesis of other PGs at the wound site also contribute to ECM repair processes in the CNS ECM. These PGs include NG2 [242,243], phosphacan [244], versican, particularly the V2 isoform [245], neurocan [246] and brevican [245]. Upregulation of chondroitin-6-sulphotransferase activity has also been shown at sites of corticol injury, leading to increased chondrotin-6-sulphation in PGs resident at the defect site [247]. Astrocytes treated with TGFα or TGFβ to mimic an injury response, upregulate Sdc-1 and HS2ST activity correlating with increased staining with the phage Ab AO4B08, which identifies high-sulphation HS glycoforms [248]. The injured adult rat brain displays an increased level of HS-PGs around defect sites, and increased 2-O-sulphation of HS side chains in Sdc-1 produced by astrocytes [249]. These two examples demonstrate how the GAG side chain sulphation of PGs is of importance as a functional determinant in neurobiological repair processes [8,10,250,251,252].

Macrophages have roles in the clearance of myelin debris following stroke and pericytes also undertake fibrotic responses which are important in the functional recovery of the BBB following ischaemic stroke [247]. Platelet-derived growth factor receptor β (PDGFRβ)-expressing pericytes are responsible for post-stroke fibrotic repair response [253,254], with the tissue macrophages and pericytes acting in a coupled manner to effect tissue repair [247] and the promotion of functional recovery of these tissues. Perlecan, a normal component of the BBB basement membrane, has regulatory roles over fibrosis in many tissues [141]. Perlecan is susceptible to proteolysis, particularly in domains IV and V [13]. Perlecan domain V is present in brain tissues at appreciable levels following stroke, where it acts as a PG in its own right, displaying interactive properties with integrin α5β1 that promote pericyte migration. This enhances PDGF-BB-induced phosphorylation of PDGFRβ, Src homology region 2 domain-containing phosphatase-2 and focal adhesion kinase supporting the repair of the BBB following ischaemic stroke [13]. Perlecan domain V has neurogenic, neuroprotective [255] properties and modulates astrogliosis [256], contributing to the functional recovery of brain tissues following ischaemic stroke [257]. While the ECM is a mechanically supportive scaffold for cell attachment and matrix deposition during tissue expansion and development, it is also a source of instructive cues that direct the migration of neuroprogenitor cells that form neural networks. The ECM also has instructive properties over angiogenic events that lead to development of the neural microvasculature [258]. Perlecan is one ECM component that supports angiogenic development through its adhesive properties for vascular cell populations, and through the growth factors it sequesters and presents to receptors to promote cellular proliferation and differentiation that promotes angiogenesis required for tissue repair [61]. FGF-binding protein 1 (FGFBP1) is a perlecan ligand and a secreted chaperone protein that mobilises stored ECM FGFs, aiding in their presentation to their cognate cellular receptors [59]. FGFBP-1 promotes the development of the BBB by regulating collagen IV deposition and maintaining Wnt/β-catenin signalling in the cell populations that undertake angiogenic development [259]. Disruption of Wnt/β-catenin signalling contributes to the breakdown of the BBB, leading to functional impairment in neural processes in Alzheimer’s disease [260]. Thus, FGFBPs modulate FGF signalling in neural development, angiogenesis [261] and wound healing [262].

Fibrinogen binds specifically to HA [263,264] and such interactions are important in fibrin clot formation during the earliest stages of the healing of vascularised wounds [265]. Astroglia and neurons express the Aα, Bβ and γ fibrinogen chains. Expression of these fibrinogen chains is elevated following subarachnoid haemorrhage, suggesting that these may have roles to play in tissue repair following traumatic injury [266]. Fibrinogen regulates sub-ventricular neural stem/progenitor cells that contribute to brain repair following TBI. HA and perlecan in this niche environment also promote neuron proliferation and differentiation through FGF-2 cell signalling [7,8,9]. Fibrinogen is a specific ligand for SVZ niche HA and triggers astrogliogenesis following corticol brain injury through BMP signalling [267,268]. Fibrinogen inhibits neuronal differentiation in the SVZ niche while it promotes astrogliogenesis via activation of BMP cell signalling. Genetic and pharmacologic interventions that deplete fibrinogen levels in the SVZ also reduce the generation of astrocytes after cortical injury and this reduces lesion scar formation. Thus, fibrinogen is a regulator of astrogenesis in the SVZ niche following TBI [269].

## 12. ECM Proteoglycans and Their Roles in Neural Network Modulation of Neuroregulatory Receptors and the Provision of Instructive Regulatory Cues to Neurotrophic Factors

As already discussed, the ECM is pivotal to CNS development and facilitates cell migration, axonal growth, myelination, dendritic spine formation and synaptic plasticity, acting as a permissive or non-permissive substrate directing axonal navigation. However, the ECM also modulates guidance cues provided by netrin, Eph/ephrin family members, Slit–Robo, semaphorin and Reln that guide axonal navigation [130,270,271,272,273,274,275,276,277,278,279,280,281,282,283,284,285,286]. HSPGs and CSPGs have roles in the dimerization/activation of many of these neuroregulatory molecules or may be integral interactive components at the cell surface. PGs are highly interactive through their GAG side chains and may also elicit specific core protein-mediated interactions affecting migrating cells directly or modulating secreted guidance cues. Specific PGs also play specific roles in the assembly of the microvasculature basement membrane, BBB, NMJ and specialised extracellular structures such as PNNs.

## 13. The CNS Is under Tension

**Figure 6:**
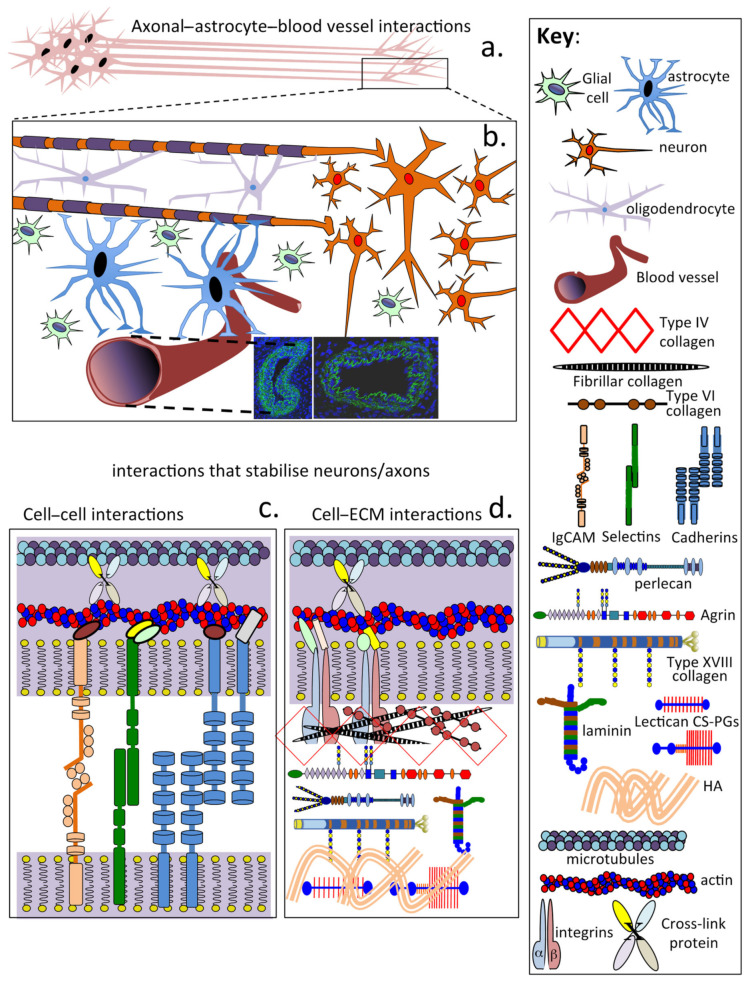
Schematic depictions of neuron axonal bundles (**a**) and the cellular and ECM interactions which provide tensile strength to neural tissues showing co-operative interactions with oligodendrocytes, astrocytes and microvascular structures (**b**). Cell–cell contacts through interactions between IgCAMs, selectins and cadherins and intracellularly with the actin and tubulin components of the cytoskeleton contribute to cell–cell adhesion (**c**). Cell–ECM interactions involving lattice-forming collagens, HS-PGs and laminin, CS-PGs and HA and tenascin-C also contribute towards the stabilization of neural architecture (**d**).

Mechanical tension along the length of axons, dendrites and glial processes is a major contributor to morphogenetic change throughout the nervous system. Tension-based morphogenesis is a conceptually simple and general hypothesis based on physical forces that help shape all living things [287]. In the cerebral cortex, tension along axons in the white matter results in the cortex folding in a characteristic species-specific pattern. In the cerebellum, tension along parallel fibres explains why the cortex is highly elongated but has a folded convoluted morphology [288]. Cells sense changes in their mechanical environment and as a consequence promote alterations and adaptations in tissue structure and function [289]. Mechanical morphogenesis is not limited to tensional and weight-bearing tissues such as cartilage and tendon. Mechanical stimuli regulate such fundamental processes as cell division and differentiation and determine tissue forms, including the CNS/PNS. Figure 6 shows the many components that contribute to the stabilisation of axons and the CNS ECM.

### Cytoskeletal and Extracellular Matrix Protein Networks Maintain Neuronal Network Architecture in the Mature CNS/PNS

Neuronal axonal extensions are the longest structures in the human body, requiring stabilisation and protection from external compressive and tensional stresses generated by extracellular skeletal components and internally within neural structures [290].

In the mature CNS/PNS, two protein networks stabilise neural/axonal network architecture, the internal cytoskeletal proteins and the cell-associated ECM (Figure 6). The cytoskeletal network consists of filamentous proteins such as actin, microtubules and neurofilaments, which are cross-linked to one another by cross-linking proteins. The actin cytoskeleton and intermediate filaments resist tensional forces, and microtubules form rigid intraneural structures that resist compressive loads. The assembly of this cytoskeletal network parallels the ECM adjacent to the neuron. Neighbouring cells in proximity to the neuron (oligodendrocytes, astrocytes and glial cells) also provide stabilisation to axonal structures through cell adhesion proteins (IgCAM, selectins, cadherins and integrins) and interactions with ECM PGs and structural glycoproteins of the perinodal ECM. Furthermore, astrocytes also provide interconnections through foot-like extensions between neural networks and the closely associated CNS/PNS microvasculature. The perinodal ECM also protects the myelin sheath from trauma. The myelin sheath around axons has essential roles to play, ensuring high neuronal signal conduction velocities are maintained and efficient neuronal signalling occurs. The myelin sheath also insulates neurons from adjacent neurons travelling in the same tract, preventing spurious signal cross-talk between adjacent neurons and dissipation of signal intensity, and also ensures specificity in neuronal signalling to its precise target site(s). Neural cells contain intracellular structural components that dictate cell shape, cellular attachment and regulation of cellular motility. These include microfilament and microtubular structures crosslinked to the actin cytoskeleton and a myriad of adaptor proteins that modify cytoskeletal architecture and their mechanical supportive properties. These intracellular networks in turn are interconnected to extracellular protein networks and adjacent cells through cell adhesive molecules including IgCAM, integrins, selectins, cadherins, transmembrane PGs and structural glycoproteins. Some selectivity in binding of these cell adhesive proteins is evident in neural networks, e.g., tenascin binds to integrins but not L1 IgCAMs, CS-PGs bind L1 IgCAMs but not integrins and focal adhesion complexes bind to integrins but not to cadherins. Cell surface interactions are important since they transmit mechanical forces into and out of cells and regulate cell signalling and the homeostasis of tissues. These intra- and extracellular tensile and compressive load-bearing architectural elements in neural systems are networked and may act as a mechanosensory system that independently regulates neural behaviour in response to external loading during traumatic brain injury [290]. The tensegrity theory proposed by Ingber (2003) [291,292] offers an explanation of how cells sense and respond to exogenous forces at the molecular level based on the architecture of their intra- and extracellular tensile and weight-bearing structural elements [293,294,295,296].

A number of ECM components support neural basal laminas, including laminins, agrin, perlecan, collagen XVIII and glycoproteins such as WARP and SPARC. PRELP and elastic microfibrils also anchor perlecan in basement membranes. Sparse contributions from fibrillar collagens I, III and V, and from lattice-forming type VI and network-forming type IV collagens, also contribute to basal membrane assembly in some nerve locations. Some neurons are also surrounded in protective perineuronal nets assembled from members of the CS-PG lectican family, tenascin-R, the HAPLN2 link protein family and HA. Phosphacan is also a component of PNNs. Laminin G-like (LG) modules in laminin, perlecan and agrin mediate stabilising ECM interactions that are crucial to basement membrane assembly and nerve cell function [297,298,299,300,301]. Perlecan contains three LG domains, and as previously mentioned, displays neurogenic and neuroprotective properties and promise in the repair of the BBB following ischaemic stroke and in the treatment of vascular dementia [255,256,257,302,303,304,305,306]. Agrin and collagen XVIII also contain multiple laminin-G domains which are interactive with other basement membrane components. The NC1 domain of collagen XVIII binds to perlecan, laminin-1, nidogen-2 and fibulin-2, while the C-terminal endostatin domain also interacts with perlecan. The glycan components of α-dystroglycan (αDG) also interact with structural glycoproteins and proteoglycans of basement membranes bearing laminin-G domains, such as laminin, agrin, perlecan, collagen XVIII, neurexin and NG2 proteoglycan (CSPG4). Matriglycan is assembled on αDG glycan chains and further extends the interactive properties of αDG in synaptic membrane organisation in basal structures in motor neuron endplates in the NMJ.

## 14. Perineuronal Nets and Related Neural Structures

**Figure 7:**
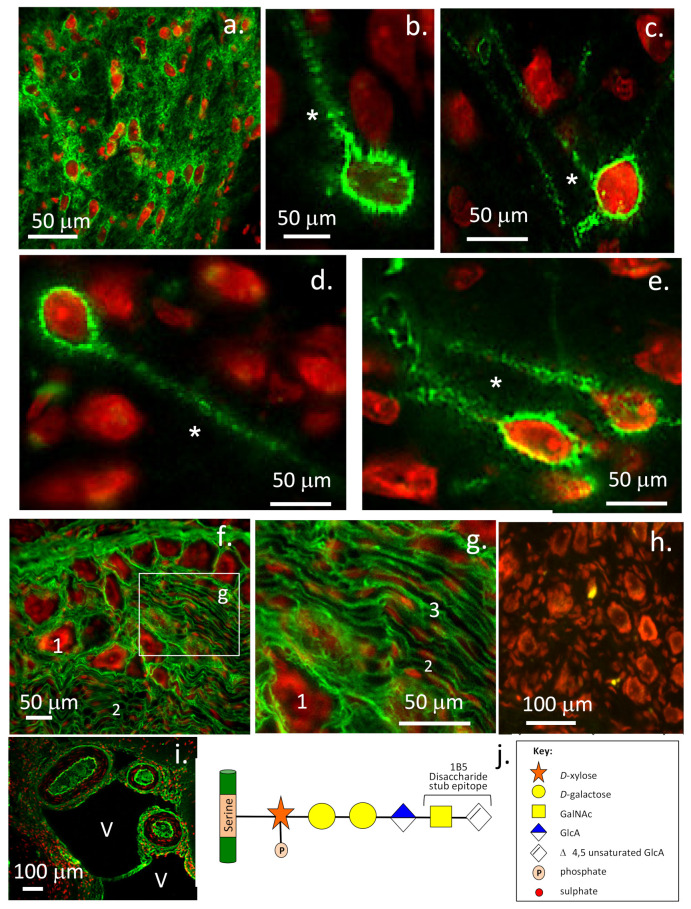
Immunolocalisation of the lectican CS-PGs using monoclonal antibody 1B5. The lectican PGs form diffuse assemblies in the brain ECM between the perineuronal nets (PNNs), as well as denser ECM in the PNNs. Lectican localisation is prominent in the cerebrum and pre-frontal cortex (**a**), but is less prominent in the PNNs of the sub-ventral cortex (**b**–**e**). Lectican PGs are also prominent components of neural tissue in the dorsal root ganglion surrounding the larger sensory neurons (1) and smaller satellite cells (2), as well as lamellar neural structures (3) (**f**,**g**) and ventricle-associated cerebral vessels in the arachnoid mater (**i**). Speckled 1-B-5 +ve dendritic processes typical of fractone stem cell niches are labelled with an asterisk (*). A negative control tissue section of the dorsal root ganglion is also shown (**h**). A schematic of the epitope identified by monoclonal antibody 1B5 is depicted in (**j**). This 1B5 epitope is generated by pre-digestion of tissue sections with chondroitinase ABC (0.1 U/mL) for 1 h at 37 °C in Tris-HCl pH 7.2. Images supplied by Prof B. Caterson, University of Cardiff, Cardiff, UK. Images © Caterson and Hayes 2012.

While lectican PGs are prominent components of PNNs, examination of neural structures in dorsal root ganglion tissues also shows that the lectican PGs are prominent components of these tissues (Figure 7). PNNs are neuroprotective condensed ECM structures composed of CS-PGs; particularly aggrecan, but other lectican CS-PGs may also be present. Phosphacan, HA, HAPLN2 and tenascin-C are also PNN components. PNNs surrounding neurons are prevalent in the mature CNS/PNS (Figure 7a). A similar structure, the perinodal ECM, surrounds the axonal nodes of Ranvier appearing when myelination is completed; this acts as a protective ion diffusion barrier. PNNs are variably distributed in different brain regions. The somatosensory frontal lobes of the cerebral cortex have a particularly high density of PNNs (Figure 7a). However, PNNs are sparsely distributed in the sub-ventricular and sub-granular dentate gyrus regions of the brain (Figure 7b–e). These are important regions in the adult brain that are responsible for the generation of neuroprogenitor stem cell populations in niche structures that have been termed fractones [307]. Ependymal cells lining the ventricles interact with a reserve of neuro-progenitor stem cells to produce this stem cell niche. Fractones have been described as stem-like or bulbous projections extending from neurons that do not communicate with the microvasculature and often have a speckled appearance [308]. A lower power view demonstrates a number of neurons surrounded by 1B5 +ve CS-PGs labelled by an asterisk, which may represent a fractone. Further immunolocalisations to demonstrate laminin and perlecan surrounding the fractone need to be shown to confirm this possibility. However, if neural perlecan is present as a hybrid HS/CS proteoglycan, as it is in many tissues, then perlecan may also contain 1B5 +ve CS side chains and is thus actually imaged in this figure along with the lectican CS-PGs. Confirmatory immunolocalisations with a range of ECM antibodies are warranted. A large range of antibodies are available to immunolocalise ECM niche components and stage neuronal development in both normal and neurodegenerative neural tissues [309,310].

ECM proteins promote growth factor access and activity in neural stem cell niches [307]. HSPGs (perlecan) and laminins are components of these fractone structures [311]. Fractone-associated HS-PGs bind growth factors and regulate neural stem cell proliferation [311]. Reduced N-sulphated HS levels have been observed in BTBR T+tf/J mice: a strong model of autism demonstrating the functional importance of these entities [311]. Perlecan has roles in growth factor sequestration in these niche structures [312] and their delivery in the differentiation of stem cells [313]. Evidence is emerging that fractones are dynamic functional entities that are altered in autism. Similar structures have been found in β-amyloid plaques in AD patients where they may result in growth factor imbalances that impair neurogenesis and lead to neural inflammation [307]. Fractone ECM structures in the neural stem cell niche influence neural stem and progenitor cell formation, proliferation and/or maintenance [314]. Ependymal cells are the source of laminin α5-containing fractone bulbs. Deletion of laminin α5 from ependymal cells results in a 60% increase in niche cell proliferation, indicating that laminin α5 modulates the proliferative status of the neural stem cell niche. The C-terminus of the five laminin α chains are key to laminin signalling and are crucial for pluripotent stem cell survival and self-renewal in vitro [314,315] and inhibit stem cell proliferation in vivo. This regulation of neuroprogenitor niche cells is believed to be due to perlecan, which interacts with laminin in the niche environment and with FGF-2 to promote niche cell proliferation [59], and with BMP-4 and BMP-7 to inhibit niche cell proliferation. The prominent immunolocalisation of 1B5 +ve CS-PGs in neural structures in the dorsal root ganglia, the lining tissues of the ventricles and large cerebral vessels and arachnoid mater further extends the distribution of these PGs in neural tissues.

## 15. Concluding Remarks

The ECM of the CNS/PNS is uniquely dominated by GAGs, particularly HA. Supportive fibrillar and sheet-like collagen and elastic networks only occur in peripheral regions in membranous structures, such as the meninges, and in specialised assemblies in the NMJ, BBB and brain microvasculature.

- CNS/PNS PGs occur as extracellular, cell-associated and intracellular proteins with diverse and specific properties styled for specific tissue and niche environments to control cellular behaviour not only in development, but also in repair processes;
- The GAG side chains of PGs are important instructive entities that direct cellular behaviour during development, repair and function of the CNS/PNS;
- An extremely diverse range of GAG structures on neural PGs provide a library of modulatory components that can be specified for precise tissue- and niche-specific environments, modifying cellular behaviour in the CNS/PNS;
- Specific modifications in PG core proteins and GAG side chains provide unique instructive properties;
- CNS/PNS ECM changes in composition and assembly in neurological disorders correlate with changes in social interaction and cognitive learning and represent targets for therapeutic intervention;
- Lectican CS-PGs have well-known protective roles in the condensed PNN matrix but are also major components of neural structures in the dorsal root ganglia, fractone stem cell niches in sub ventricular and dentate gyrus of the hippocampus and muscular cerebral vessels associated with the ventricles of the brain;
- The ECM guides neural network assembly and the neurovascular unit. Embedded vascular growth and trophic factors direct neural cell populations through vessel–astrocyte foot processes, facilitating cross-talk with neural cells.

## Data Availability

All data is provided within the cited references.

## Abbreviations

Ab	Amyloid-beta
ACAN	Aggrecan
AD	Alzheimer’s disease
ADAMTS	A Disintegrin and Metalloproteinase with Thrombospondin motifs
Akt	Protein kinase B, “AK” derived from AK mice, “t” from thyoma
ALS	Amyotrophic lateral sclerosis
APP	Alzheimer Amyloid Precursor Proteoglycan
BBB	Blood–brain barrier
BEHAB	Brain-enriched hyaluronan-binding (Brevican)
BGAN	Biglycan
CALEB	Acidic leucine-rich EGF-like domain-containing brain protein
CLEVER	Common lymphatic endothelial and vascular endothelial receptor-1
CNS/PNS	Central nervous system/peripheral nervous system
CS	Chondroitin sulphate
CUB	Complement C1r/C1s, Uegf, Bmp1
DEC	Decorin
DS	Dermatan sulphate
α-DG	α-dystroglycan
ECM	Extracellular matrix
EGF	Epidermal growth factor
pEGFR	Phosphorylated EGF receptor
EMILIN	Elastin microfibril interface-located protein
pERK	Phosphorylated extracellular signal-regulated kinase
FMOD	Fibromodulin
G1, G2, G3	Globular domains G1, G2, G3
GAG	Glycosaminoglycan
GHAP	Glial HA-binding protein
HA	Hyaluronan
HABP2	Hyaluronan-binding protein-2
HAI-1, 2	Hepatocyte growth factor activator inhibitor type 1, 2
HAPLN2	Hyaluronan link protein-2
HARE	HA Receptor for Endocytosis
HC	Heavy chain
HS	Heparan sulphate
HNK	Human natural killer trisaccharide
IHABP4	Intracellular HA-binding protein-4
ITI	Inter-α-trypsin inhibitor
KER	Keratocan
KS	Keratan sulphate
*LARGE*	Glycosyltransferase-like protein encoded by the *LARGE* gene, a gene associated with muscular dystrophy
LAYN	Layilin
LG-1, 2, 3	Laminin-G domains-1, 2, 3
Lrp4	LDL Receptor-Related Protein 4
LUM	Lumican
LYVE-1	Lymphocyte vascular endothelial cell-1
MAP1B	Microtubule-Associated Protein 1B
MMP	Matrix metalloprotease
NCAM	Neural cell adhesion molecule
NG2	Neural glial proteoglycan-2
NGC	Neuroglycan-C
NGF	Nerve growth factor
NMJ	Neuromuscular junction
PEP-1	A synthetic HA-binding peptide, GAHWQFNALTVR
PGs	Proteoglycans
PI3K	Phosphatidylinositol-3-kinase
PNNs	Perineuronal nets
PRELP	Prolargin, proline/arginine-rich and leucine-rich repeat protein
PTP	Protein tyrosine phosphatase
RHAMM	Receptor for HA-mediated motility
RPTP	Receptor protein tyrosine phosphatase
SARCO	Sarcoidosis
SC	Spinal cord
SCI	Spinal cord injury
SHAP	Serum hyaluronan protein
SPACRCAN	Sialoprotein associated with cones and rods proteoglycan
STAB	Stabilin
SV2	Synaptic vesicle proteoglycan-2
TFPI	Tissue factor proteinase inhibitor
TGF-β	Transforming growth factor-β
TLR-4	Toll-like receptor 4
TNFα	Tumour necrosis factor-α
TSG-6	Tumour necrosis factor inducible gene 6 protein
VEGF	Vascular endothelial cell growth factor
Wnt	Wingless/int
